# Using Two Detection Methods to Observe the Changes and Significance of Free Light Chain in Serum and Urine in Patients with Renal Insufficiency

**DOI:** 10.1155/2022/5536199

**Published:** 2022-03-29

**Authors:** Lengnan Xu, Ban Zhao, Ying Sun, Songlan Wang, Xianguang Chen, Yonghui Mao

**Affiliations:** Department of Nephrology, Beijing Hospital, National Center of Gerontology, Institute of Geriatric Medicine, Chinese Academy of Medical Sciences, China 100730

## Abstract

**Background:**

Free light chains *κ* and *λ* (FLC *κ*, FLC *λ*) are of great significance in diagnostic and monitoring monoclonal gammopathy. Freelite and N-Latex methods are two common monitoring methods at present. But the two meanings are not completely equivalent, especially for patients with renal insufficiency. We analyzed the changes of serum and urine FLC in renal insufficiency patients without monoclonal gammopathy and the clinical significance of these changes.

**Methods:**

This study is an observational study. Patients ≥ 18 years old, who met the diagnostic criteria of chronic kidney disease (CKD), excluding monoclonal gammopathy, were selected. Fasting serum and 24-hour urine were taken to detect serum FLC *κ*, serum FLC *λ*, SCr, serum *β*_2_-microglobulin, urinary FLC *κ*, urinary FLC *λ*, urinary *α*_1_-microglobulin, and urinary *β*_2_-microglobulin.

**Results:**

There was a good correlation between the two methods for determining serum/urinary FLC. No matter serum or urine, FLC showed a good correlation with renal function by the N-Latex method, but not by the Freelite method. Under the N-Latex method, FLC *κ*/*λ* remained stable, which was basically within the reference range of healthy people and was not affected by renal function. There was a good correlation between FLC detected by N-Latex and microglobulin in serum and urine.

**Conclusion:**

When the concentration of FLC is low, the N-Latex method is more recommended to monitor FLC. The FLC measured by the N-Latex method is more closely related to renal function. The ratio of FLC *κ*/*λ* determined by the N-Latex method remained stable within the recommended range.

## 1. Introduction

Serum free light chains *κ* and *λ* (FLC *κ*, FLC *λ*) are one of the response criteria for diagnostic and monitoring monoclonal gammopathy [1–3]. The incidence of multiple myeloma (MM) in China is 0.6/100000. In Chinese mainland, Hong Kong, and Taiwan, 24%, 19.7%, and 30.8% of MM patients had renal insufficiency at the time of diagnosis of MM (defined as serum creatinine (SCr) ≥ 176.8 *μ*mol/L) [4]. Impaired renal function will affect the metabolism of FLC and lead to the increase of serum FLC, which is not conducive to the diagnosis and monitoring of monoclonal gammopathy. In patients with monoclonal gammopathy complicated with renal insufficiency, the significance of FLC is more unclear. The false positive of FLC may be due to the decrease of renal tubular reabsorption and the increase of polyclonal light chain excretion from urine; when the renal function decreases further, it will appear false negative, and the deposition of light chain will lead to renal tubular blockage and further aggravate renal failure. The significance of urinary FLC is not clear so far, and it is more affected by renal function (glomerular filtration and renal tubular reabsorption).

In 2001, a method for the detection of FLC was developed for the first time [5]: Freelite assay is an immune turbidimetric method, which uses polyclonal antibodies against hidden epitopes of FLC and recognizes only FLC that is not related to the heavy chain. In 2011, another N-Latex FLC method using monoclonal antibodies was developed [6]. The two detection methods are not completely equivalent. In the case of renal insufficiency, the results of the two methods are even more different.

Through this study, we intend to understand the changes of serum and urine FLC in patients with renal insufficiency without monoclonal gammopathy and the clinical significance of these changes.

## 2. Subjects and Methods

### 2.1. Subjects

This study is an observational study. Since May 1, 2019, patients were continuously enrolled in the group according to the order in which they were admitted to the ward of the Department of Nephrology of Beijing Hospital. According to K/DOQI guidelines [7], patients were divided into 5 groups according to their eGFR levels (calculated based on the CKD-EPI equation of serum creatinine, stage 1: eGFR ≥ 90 mL/min/1.73 m^2^; stage 2: 60 mL/min/1.73 m^2^ ≤ eGFR < 90 mL/min/1.73 m^2^; stage 3: 30 mL/min/1.73 m^2^ ≤ eGFR < 60 mL/min/1.73 m^2^; stage 4: 15 mL/min/1.73 m^2^ ≤ eGFR < 30 mL/min/1.73 m^2^; and stage 5: eGFR < 15 mL/min/1.73 m^2^). There were 50 patients in each group, and the number of patients in the group was stopped after the number of patients reached the standard.

The inclusion criteria were as follows: (1) age ≥ 18 years old; (2) it meets the diagnostic criteria of chronic kidney disease (CKD) which was diagnosed by kidney damage for ≥3 months as defined by structural or functional abnormalities of the kidney, with or without decreased GFR, or GFR < 60 mL/min/1.73 m^2^ for ≥3months, with or without kidney damage [7]. The exclusion criteria were as follows: (1) complicated with hematological tumors or solid tumors, such as MM, monoclonal gammopathy of undetermined significance, monoclonal gammopathy of renal significance, and renal cell carcinoma; (2) diagnosed as acute kidney injury, which was diagnosed by an increase in serum creatinine by ≥0.3 mg/dL (≥26.5 *μ*mol/L) within 48 h or an increase in serum creatinine to ≥1.5 times baseline within the previous 7 days or urine volume ≤ 0.5 mL/kg/h for 6 h [8]; (3) complicated with acute infection, acute myocardial infarction, acute heart failure, shock, and other acute complications; (4) use of nephrotoxic drugs and additional protein supplements (intravenous or oral); and (5) need renal replacement therapy or have entered MHD.

This study has been approved by the Ethics Committee of Beijing Hospital (Approval Letter No. 2019BJYYEC-062-01).

### 2.2. Study Methods

Fasting serum and 24-hour urine were taken from each patient in the same period. The following tests were performed: serum FLC *κ*, serum FLC *λ*, SCr, serum *β*_2_-microglobulin, urinary FLC *κ*, urinary FLC *λ*, urinary *α*_1_-microglobulin, and urinary *β*_2_-microglobulin. Serum levels of creatinine were determined by an enzymatic method (Hitachi 008AS Automatic biochemical analyzer, Tokyo, Japan). Other items were tested by Siemens BN II. FLC in serum and urine were detected by the Freelite™ assay (IMMAGE 800, the Binding Site Ltd, Birmingham, UK) and the N-Latex FLC assay (Siemens BN II, Siemens, Germany) at the same time.

We chose to use the reference ranges proposed in the literature [5, 6, 9]: N-Latex: FLC *κ*6.7-22.4 mg/L; FLC *λ* 8.3-27.0 mg/L, FLC *κ*/*λ*0.31-1.56; Freelite: FLC *κ* 3.3.0-19.40 mg/L; FLC *λ* 5.71-26.30 mg/L, FLC *κ*/*λ* 0.26-1.65 (0.37-3.10 mg/L if eGFR < 60 mL/min/1.73 m^2^).

### 2.3. Statistical Methods

IBM SPSS 22.0 were used for data analysis. First, we verified whether the continuous variables belong to normal distribution. For normal distribution variables, mean ± standard deviation was used; single-factor ANOVA was used to compare different groups; Pearson was used to test the correlation between data.

## 3. Results

### 3.1. Demographic Data and General Clinical Conditions

A total of 250 patients were selected, including 145 males (58.0%) and 105 females (42.0%). The average age was 58.93 ± 15.75 years old, of which 58.18 ± 16.59 years old in males and 60.10 ± 14.35 years old in females. 95 patients (38.0%) suffered from primary glomerulopathy (diagnosed by renal biopsy), 73 patients (29.2%) with diabetic nephropathy, 23 patients (9.2%) with hypertensive nephropathy, 14 patients (5.6%) with secondary glomerulonephritis, 8 patients (3.2%) with chronic interstitial glomerulonephritis, 7 patients (3.5%) with obstructive nephropathy, 6 patients (2.4%) with renal artery stenosis, and 24 patients (9.6%) with renal artery stenosis.

### 3.2. Relationship between Serum FLC and Renal Function

**Figure 1:**
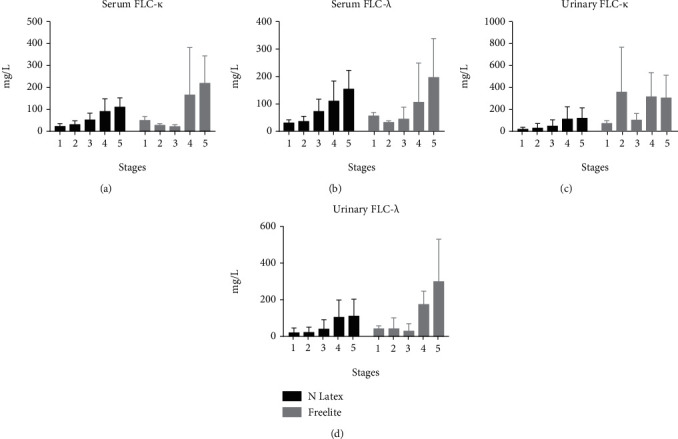
Expression of serum and urinary FLC detected by two methods in different renal functions: (a) serum FLC *κ*; (b) serum FLC *λ*; (c) urinary FLC *κ*; (d) urinary FLC *λ*. FLC: free light chain.

**Figure 2:**
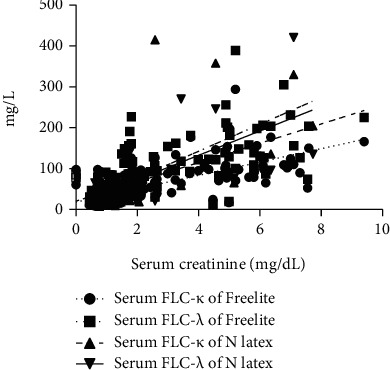
The relationship between serum FLC detected by two methods and serum creatinine. FLC: free light chain.

Using N-Latex to detect FLC, with the aggravation of renal function injury, the concentration of serum FLC increased step by step (Figures 1(a) and 1(b)), which was significantly correlated with SCr (FLC *κ*: *R* = 0.703, *P* ≤ 0.001; FLC *λ*: *R* = 0.505, *P* = 0.001, Figure 2). However, using Freelite to detect, it was found that there was no close relationship between FLC and renal function (FLC *κ*: *R* = 0.463, *P* = 0.071; FLC *λ*: *R* = 0.500, *P* = 0.051, Figure 2); and the regular changes of Freelite method with renal function are not seen in Figures 1(a) and 1(b), with abnormally high increases in stages 4 and 5. Using the N-Latex method, the ratio of serum FLC *κ*/*λ* was within the reference intervals (in subjects with normal renal function, median value 0.86, minimum-maximum value 0.31-1.56), while under the Freelite method, the ratio was greatly affected by renal function, and the ratio was significantly higher than the normal range in stage 4 (0.37-3.10) (Table 1). There was a good correlation between the two methods for determining serum FLC (FLC *κ*: *R* = 0.789, *P* ≤ 0.001; FLC *λ*: *R* = 0.666, *P* = 0.001).

### 3.3. Relationship between Urinary FLC and Renal Function

As can be seen from Figures 1(c) and 1(d), the urinary FLC showed a ladder-like increase with the aggravation of renal function damage by the N-Latex method, but the correlation was not strong (*κ*: *R* = 0.188, *P* = 0.003; *λ*: *R* = 0.325, *P* ≤ 0.001). There was no clear correlation by the Freelite method. The level of urinary FLC *κ*/*λ* obtained by the N-Latex method was relatively stable, but only phase 2 and stage 3 were within the recommended range of the manual (1.4-6.2, 2.5-97.5 percentile). However, the ratio obtained by the Freelite method was greatly affected by renal function, in which stage 2 and stage 3 were significantly higher than the reference range (0.461-4.0) (Table 1). There was a good correlation between the two methods for determining urinary FLC in all stages (FLC *κ*: *R* = 0.946, *P* ≤ 0.001; FLC *λ*: *R* = 0.827, *P* = 0.001).

### 3.4. The Relationship between FLC and Other Markers

Microglobulin can freely pass through the glomerulus and reabsorb through the renal tubule. Therefore, serum microglobulin reflects the ability of glomerular filtration, and urinary microglobulin reflects the state of early renal tubular injury. As can be seen from Table 2, FLC measured by the N-Latex method had a good correlation with microglobulin and can be used as a marker of renal injury. However, the correlation between FLC and microglobulin determined by the Freelite method is poor, which cannot fully reflect the damage of the kidney.

## 4. Discussion

Each plasma cell produces one of the five heavy chains and *κ* or *λ* light chains. After forming a complete immunoglobulin molecule, about 40% of the light chains remain into the circulation. The production speed of *κ* chain is about twice as fast as that of *λ* chain, and FLC *κ* is often a monomer, while FLC *λ* is easy to form a dimer. In normal individuals, serum FLC is rapidly cleared and metabolized by the kidney. Monomer FLC (*κ* chain) is cleared at 40% glomerular filtration rate within 2-4 hours. The dimer FLC (*λ* chain) is cleared at 20% glomerular filtration rate within 3-6 hours. FLC is completely through the glomeruli, degraded in the proximal renal tubules, and then absorbed or excreted in the form of fragments. The scavenging rate of FLC *κ* is 3 times faster than that of dimer FLC *λ*, so the concentration in serum is only about half of that of FLC *λ* [10].

In patients with CKD, the clearance rate of polyclonal FLC decreases and the serum concentration increases. Mild to moderate renal failure prolongs the half-life of both FLC from hours to days, so the concentration of FLC in serum increases by 20 times or more, but *κ*/*λ* changes only in a small range—it can be used to distinguish between monoclonal and polyclonal diseases. However, with the aggravation of renal failure, the ratio of *κ*/*λ* will increase, even as high as 3.1 [11]. The content of urinary FLC is more dependent on renal function. Due to the powerful function of renal tubules, FLC will not appear in urine under normal status. The concentration range of urinary FLC is wider than that of serum, and the variability of *κ*/*λ* is greater. Therefore, it can reflect small changes in renal processing function, urine dilution function, and FLC changes.

With the availability of the Freelite method, the determination of FLC has been incorporated into guidelines for multiple myeloma and related diseases [1–3]. However, the Freelite method has the limitations of poor linearity and relative inaccuracy after dilution, as well as the increased possibility of false-negative results due to excessive antigens in patients with extremely high FLC concentrations. In order to overcome these problems, N-Latex system has been developed [6]. This is a highly selective new immune scattering turbidimetry. This high selectivity can only be achieved by anti-FLC monoclonal antibodies rather than polyclonal antibodies. The method shows good performance in selectivity, sensitivity, precision, linearity, antigen excess safety, and interbatch consistency [12]. The reference range of the N-Latex system is basically consistent with that of Freelite, which is within its analyzable range.

The Freelite method is established using sheep polyclonal antibodies to detect only FLC wavelength dimers, while the N-Latex test includes several mouse monoclonal antibodies that only detected FLC monomers [13]. In the determination of polyclonal antibody samples, the two monitoring methods are highly consistent, while in the determination of monoclonal antibodies, the two methods are not very consistent. This may be related to free light chain dimerization leading to different scatter efficiency of macromolecular complexes [14].

Many researchers have compared the two different detection methods and found that the results of the two kinds of FLC have good correlation and consistency are even in patients with kidney failure. This study confirmed this finding. However, the accuracy of the two methods for FLC *κ* and *λ* is slightly different, but this difference does not seem to affect the prognosis of the patients. Therefore, researchers believe that the two detection methods are limited in clinical application and cannot be replaced with each other [15–17]. Our study found that the serum/urinary FLC detected by the N-Latex method showed a good correlation with renal function: with the aggravation of renal function damage, FLC increased step by step. While the correlation of the Freelite method was poor, CKD 1-3 stage did not change much, and CKD 4-5 stage increased sharply. It showed that there was a great interference in the determination of FLC by the Freelite method for patients with impaired renal function, especially in the advanced stage. This might be related to the dimer detected by Freelite. The process of dimer filtration through the kidney is more likely to be affected by renal function.

There may be another reason for this. When testing different reagent batches at low concentrations (e.g., when FLC excretion is unimpeded in normal renal function and both serum and urine FLC concentrations are low), the Freelite method will result in a large error in the results [18], whereas the N-Latex method will be relatively stable (less error). From a physiological point of view, FLC should be related to renal function. The N-Latex method in our results showed changes in FLC related to renal function, while the results of the Freelite method did not have much rule to follow. Therefore, the N-latex method is more recommended for monitoring FLC when FLC concentrations are low (e.g., without plasma cell disease).

In view of the influence of renal function on FLC, the ratio of urinary FLC *κ* and *λ* is of greater significance [19]. The ratio of *κ*/*λ* was determined by Freelite and N-Latex FLC, respectively. The regression line almost overlapped with the same line in the reference range of healthy control, and the best consistency was observed. In patients with highly abnormal FLC ratios (patients with renal insufficiency), greater measurement uncertainty was observed, resulting in significant differences in FLC ratios between the two methods [20]. In 2014, Dutch researchers retrospectively analyzed the changes of blood free light chain in patients with CKD and concluded that the FLC *κ*/*λ* measured by the N-Latex method had little change in patients with impaired renal function, and no FLC *κ*/*λ* exceeded the N-Latex reference limit of healthy controls. However, the FLC *κ*/*λ* determined by the Freelite method increased significantly with the increase of renal function damage [21]. It has been confirmed by many researchers that the determination of ratio by the N-Latex method has nothing to do with renal function [22]. The latest research in 2020 showed that the FLC *κ*/*λ* obtained by the Freelite method was negatively correlated with eGFR, while N-Latex was positively correlated, but they were all within the reference range of healthy people [23]. Our research also confirmed this. Under the N-Latex method, serum FLC *κ*/*λ* was within the recommended range, while under the Freelite method, the ratio was significantly higher than the normal range. The expression of urinary FLC *κ*/*λ* was less than that of N-Latex, and it was more stable than that of urine *κ*/*λ*.

In view of the fact that FLC passes freely through the glomerular filtration membrane, most of it is reabsorbed by renal tubules, which is similar to microglobulin. In our study, there was a high correlation between FLC and microglobulin measured by the N-Latex method, while the correlation was poor by the Freelite method. It also showed that the FLC measured by the N-Latex method can better reflect the state of renal disease in patients with renal insufficiency.

## 5. Conclusion

The metabolism of FLC depends on renal function. When the concentration of FLC is low, the N-Latex method is more recommended to monitor FLC. The FLC measured by the N-Latex method is more closely related to renal function. The ratio of FLC *κ*/*λ* determined by the N-Latex method is not affected by renal function and remained stable within the recommended range.

## Figures and Tables

**Table 1:** The ratio of serum and urinary FLC *κ* and *λ* detected by two methods in different stages of CKD.

	Stages	1	2	3	4	5
Serum	N-Latex	0.77 ± 0.17	0.88 ± 0.43	0.73 ± 0.15	0.83 ± 0.68	0.72 ± 0.22
	Freelite	0.94 ± 0.36	0.89 ± 0.33	0.68 ± 0.27	7.12 ± 11.29^#^	1.21 ± 0.35^#^

Urinary	N-Latex	1.01 ± 0.86	1.46 ± 0.96^#^	1.27 ± 0.71^#^	1.10 ± 0.81	1.09 ± 0.43
	Freelite	1.82 ± 0.29	12.49 ± 4.67^#^	6.32 ± 2.91^#^	2.28 ± 2.21	1.11 ± 0.14

^#^Compared with other stages, *P* < 0.05. FLC: free light chain.

**Table 2:** The relationship between FLC and microglobulin both in serum and urine by two methods.

	FLC	MG	N-Latex		Freelite	
			*R*	*P*	*R*	*P*
Serum	*κ*	*β*_2_	0.855	≤0.001	0.741	0.020
	*λ*	*β*_2_	0.534	≤0.001	0.262	0.366

Urine	*κ*	*α*_1_	0.716	≤0.001	0.638	0.089
		*β*_2_	0.376	≤0.001	0.611	0.107
	*λ*	*α*_1_	0.942	≤0.001	0.893	0.003
		*β*_2_	0.769	≤0.001	0.879	0.004

FLC: free light chain; MG: microglobulin.

## Data Availability

The data used to support the findings of this study are available from the corresponding author upon request.
